# Phylogeography and Conservation Genetics of a Distinct Lineage of Sunfish in the Cuatro Ciénegas Valley of Mexico

**DOI:** 10.1371/journal.pone.0077013

**Published:** 2013-10-10

**Authors:** Lyndon M. Coghill, C. Darrin Hulsey, Johel Chaves-Campos, Francisco J. García de Leon, Steven G. Johnson

**Affiliations:** 1 Department of Biological Sciences, University of New Orleans, New Orleans, Louisiana, United States of America; 2 Department of Ecology and Evolutionary Biology, University of Tennessee, Knoxville, Knoxville, Tennessee, United States of America; 3 Laboratorio de Genética para la Conservación, Centro de Investigaciones Biológicas del Noroeste, La Paz, Baja California Sur, Mexico; Institut de Biologia Evolutiva - Universitat Pompeu Fabra, Spain

## Abstract

The valley of Cuatro Ciénegas, an aquatic oasis located in the Mexican Chihuahuan Desert, exhibits the highest level of endemism in North America and is a Mexican National Protected Area. However, little is known about the evolutionary distinctiveness of several vertebrate species present in the Cuatro Ciénegas valley. We conducted a phylogeographic study using mitochondrial haplotypes from the centrarchid fish *Lepomis megalotis* to determine if the populations found within the valley were evolutionarily distinct from populations outside the valley. We also examined if there was evidence of unique haplotypes of this sunfish within the valley. Genetic divergence of *L. megalotis* suggests populations within the valley are evolutionarily unique when compared to *L. megalotis* outside the valley. Significant mitochondrial sequence divergence was also discovered between *L. megalotis* populations on either side of the Sierra de San Marcos that bisects the valley. Our results reinforce previous studies that suggest the organisms occupying aquatic habitats not only within Cuatro Ciénegas but also in each of the two lobes of the valley generally deserve independent consideration during management decisions.

## Introduction

Phylogeographic analyses based on molecular markers are now widely used in conservation studies to identify unique evolutionary lineages. These analyses can clarify the evolutionary context of organismal diversification especially when combined with various geological and climatic events [1]. Examination of the spatial patterns of intraspecific gene flow can also lead to the discovery of cryptic but genetically distinct populations [2-4]. In addition, molecular phylogeographies can be used to obtain a temporal context for major population subdivision and facilitate inferences of the historical forces that have produced contemporary patterns of population structure [5]. Determining the distinctiveness and age of populations especially in highly threatened habitats is essential to both managers and policy makers attempting to identify the population units most in need of conservation. Genetically identifying unique, persistent lineages of organisms can also address the impact that the loss of particular populations would have on overall biodiversity [6-8]. Within this framework, we examine the population structure and temporal divergence of long-eared sunfish populations, *Lepomis megalotis*, in a hotspot of aquatic endemicity.

**Figure 1:**
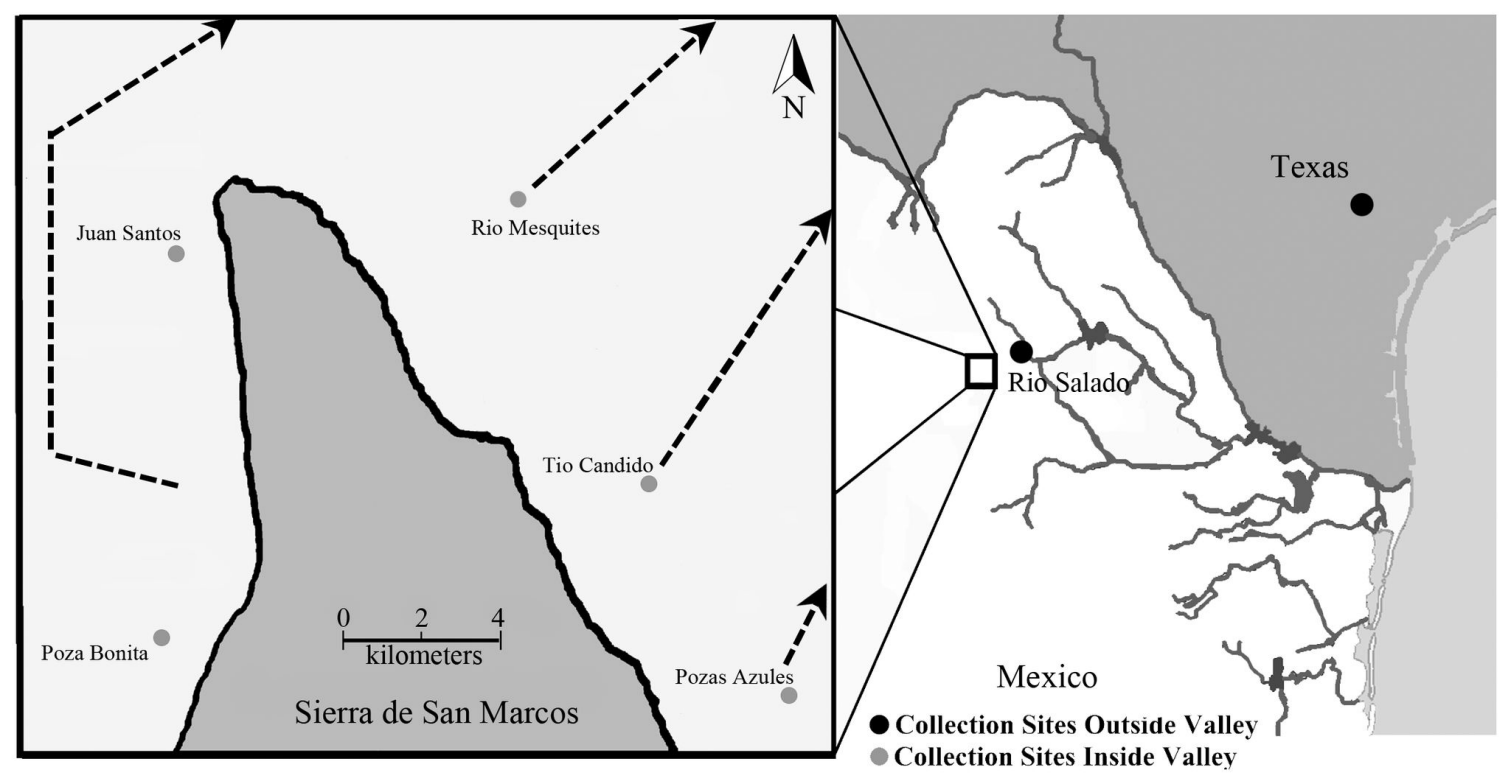
The Cuatro Ciénegas basin, Río Salado de los Nadadores, and the valley’s general location in Northern Mexico. The inset shows an enlarged diagram of the valley geography, and labels the various sampling locations with dots. Alabama sampling location is not shown.

The Cuatro Ciénegas valley exhibits the highest level of endemism in North America, but the genetic distinctiveness of many species and populations within the valley remains unclear [9]. Because of its biological uniqueness, Cuatro Ciénegas has been designated a National Protected Area by the Mexican Government, a RAMSAR site (intergovernmental treaty protected wetland) as well as an UNESCO World Heritage Biosphere Reserve [9,10]. This relatively small (~1500km^2^) intermontane valley located in the Chihuahuan desert contains numerous aquatic habitats and is home to more than 70 endemic species [10]. The valley is located in the center of an extremely arid region and virtually all of the endemic species are found within its more than 200 permanent pools, rivers, and lakes. These water bodies are also isolated into several hydrologically distinct drainages that were historically separated from aquatic connections outside of the valley [9]. The closest external drainage to the valley is the Río Salado de los Nadadores basin, but no natural aquatic connection exists between the two areas. However, several canals that carry water from the valley to agricultural land outside the valley have been constructed [6,8,9]. These man-made hydrologic connections could have provided an avenue for putatively non-endemic species such as the long-ear sunfish, *Lepomis megalotis*, to invade Cuatro Ciénegas and spread to numerous parts of the valley [11]. Alternatively, the Cuatro Ciénegas lineage of *Lepomis megalotis* could be an endemic evolutionary lineage, and like many of the valley’s other aquatic species, it could show substantial phylogeographic substructure within the valley.

Within the valley, the Sierra de San Marcos demarcates a deep genetic subdivision for several species. This mountain splits the valley into eastern and western partitions (Figure 1). The two endemic pupfish (*Cypriodon* spp.), largemouth bass within the valley (*Micropterus* spp.), one of the endemic aquatic snails (*Mexipyrgus churinceanus*), and the endemic freshwater shrimp (*Palaemonetes suttkusi*) all show patterns of geographic isolation on either side of this Sierra [6,8,12,13]. However, other species with a relatively high capacity for dispersal like the endemic box turtle (*Terrapene coahuila*) exhibit little population structure within the valley [14]. Most of the species that show high levels of population genetic structure are obligately aquatic species, and those that show little differentiation are capable of crossing small parts of dry land. However, although *L. megalotis* is restricted to aquatic habitats, it does have an extensive range outside the valley. Therefore, this sunfish might be predicted to show limited genetic structure within the Cuatro Ciénegas valley and could even exhibit little divergence between populations found inside and outside of the valley. 

*Lepomis megalotis* is one of several species that are found both within Cuatro Ciénegas and in the adjacent Río Salado drainage that ultimately drains into the Río Grande (Miller et al. 2006) (Figure 1). Like the large-mouth bass, *Micropterus salmoides*, that also occurs in both areas, *L. megalotis* could have easily been introduced into the Rio Salado or Cuatro Ciénegas due to their popularity as a game fish (Lee et al., 1980; Near et al., 2004). The native range of *L. megalotis* in North America extends from Ohio to the Gulf of Mexico [15,16], and its native range is believed to include parts of Northeastern Mexico [17]. However, no studies have examined whether populations in Mexico represent divergent entities, and the wide-ranging *L. megalotis* species complex could exhibit substantial genetic structure in many parts of its range [18]. 

**Figure 2:**
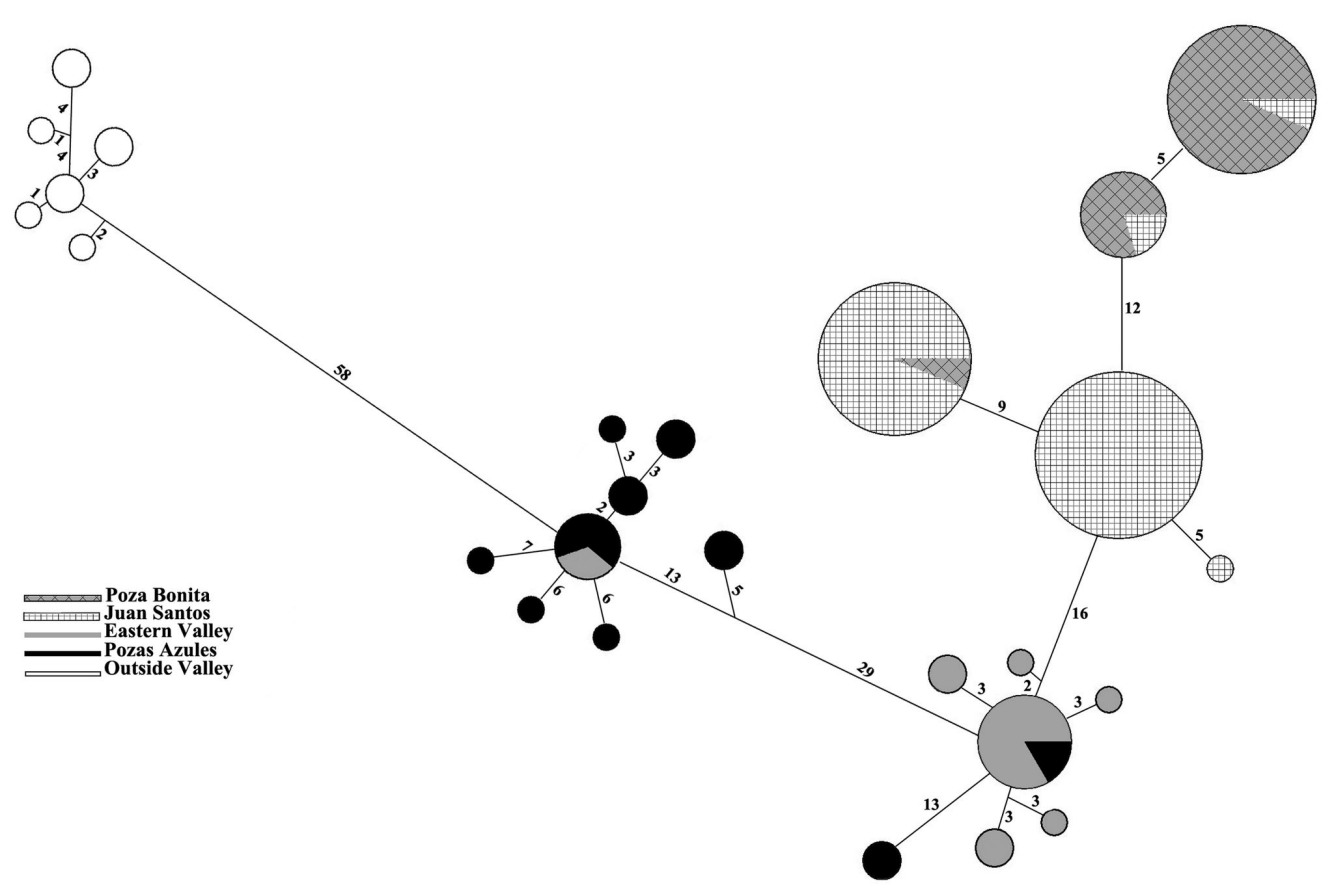
Haplotype network generated using a median-joining method. Pie graphs are proportional to the haplotype frequencies. Branch lengths are roughly proportional to the number of mutational steps between nodes. The number of steps is shown near each branch.

The primary goal of this study was to determine whether the *Lepomis megalotis* populations found within the Cuatro Ciénegas basin are genetically unique and should receive increased conservation attention. In order to investigate this idea, three specific questions were examined. First, we asked whether *L. megalotis* mitochondrial haplotypes from within the valley are highly divergent from haplotypes outside of the Cuatro Ciénegas basin. Second, we determined whether populations within the valley show phylogeographic structure. Third, we tested several gene flow models to determine whether contemporarily isolated populations of *L. megalotis* in Cuatro Ciénegas exhibit evidence of recent gene flow. 

## Materials and Methods

### Ethics Statement

This study was conducted in Mexico as a part of an international, multi-taxa study and was approved by the Mexican Government and SEMARNAT (The Ministry of Environment and Natural Resources for Mexico) which approved all field and laboratory protocols under (Permit No. N**°**DAPA/2/130409/0961 and DAN-01202). 

### Sampling and Laboratory Procedures

Samples of *L. megalotis* were collected in June 2009 and August 2010 from several sites in the Cuatro Ciénegas basin as well as several locations from outside the valley. Within the Cuatro Ciénegas basin, we sampled 6 sites that spanned the geographic breadth of the valley (Figure 1). Sample locations, sample size and GPS coordinates are given in Table 1. Samples of *L. megalotis* collected outside the valley were obtained from the Río Salado drainage directly outside of the valley and also from Texas and Alabama. In total, tissue samples from 77 individuals were examined. 

**Table 1:** List of *Lepomis megalotis* sampling localities, GPS coordinates, and sample size (n) from each locality.

**Sampling Location**	**Latitude**		**Longitude**	***n***
Juan Santos, Cuatro Ciénegas, Coahuila	26°53.859'N	102° 08.807'W		17
Poza Benito, Cuatro Ciénegas, Coahuila	26° 50.232'N	102° 08.438'W		24
Pozas Azules, Cuatro Ciénegas, Coahuila	26° 49.730'N	102° 01.683'W		11
Río Mesquites, Cuatro Ciénegas, Coahuila	26° 55.378'N	102° 6.753'W		11
Río Salado, Coahuila	27° 02.059'N	101° 43.300'W		1
Tío Candido, Cuatro Ciénegas, Coahuila	26° 52.225'N	102° 04.740'W		4
Brazos River, Texas	30° 53.016’N	95° 17.3591’W		6
Uphapee Creek, Alabama	32° 28.053’N	85° 47.059’W		4

From the left, columns show the name of the sampling locations in both Mexico and the U.S., the GPS coordinates of the sites, and the sample size examined from each location.

For all 77 individuals, we sequenced three mtDNA genes comprising 2839bp (ND2: 1047, Cytb: 1140 and COI: 652). First, DNA was extracted in the laboratory from fin tissue using Qiagen Blood and Tissue Kit (Qiagen). Primers for the three gene regions used were taken from published studies: Cytochrome *b* F: CTGCCCCCTCAAACATTTCA R: GGTTGGGGGAGAATAAGGCTAA, 53°C [19]; Cytochrome *c* oxidase subunit I, F: TCAACCAACCACAAAGACATTGGCAC, R: TCGACTAATCATAAAGATATCGGCAC, 54°C [20]; NADH dehydrogenase 2, F: CTACCTGAAGAGATCAAAAC, R: CGCGTTTAGCTGTTAACTAA, 55°C, [21]. Amplifications were carried out in a BioRAD iCycler Gradient thermocycler and conditions generally consisted of an initial denaturation step of 94 °C (2.0 min) followed by 35 cycles between 54-60 °C (30 s), and 72 °C (1.5 min). A final incubation of 72 °C for 4 min was added to ensure complete extension of products. Positively amplified DNA was then purified using an enzymatic combination of 1 μl of Exonuclease I (10.0 U/μl) and 1 μl shrimp alkaline phosphatase (2.0 U/μl) per 10μl of PCR product. Treated PCR products were sequenced at the W.M. Keck Conservation and Molecular Genetics Laboratory at the University of New Orleans using the same primers utilized for amplification. Complete gene sequences were assembled from individual reactions using the program Geneious version 5.3.6 [22]. All sequences were deposited in GenBank (KF571474-KF571702). Additional sequences for the outgroup comparison were collected from GenBank (AY517741, JN027026 and AY828969). 

**Figure 3:**
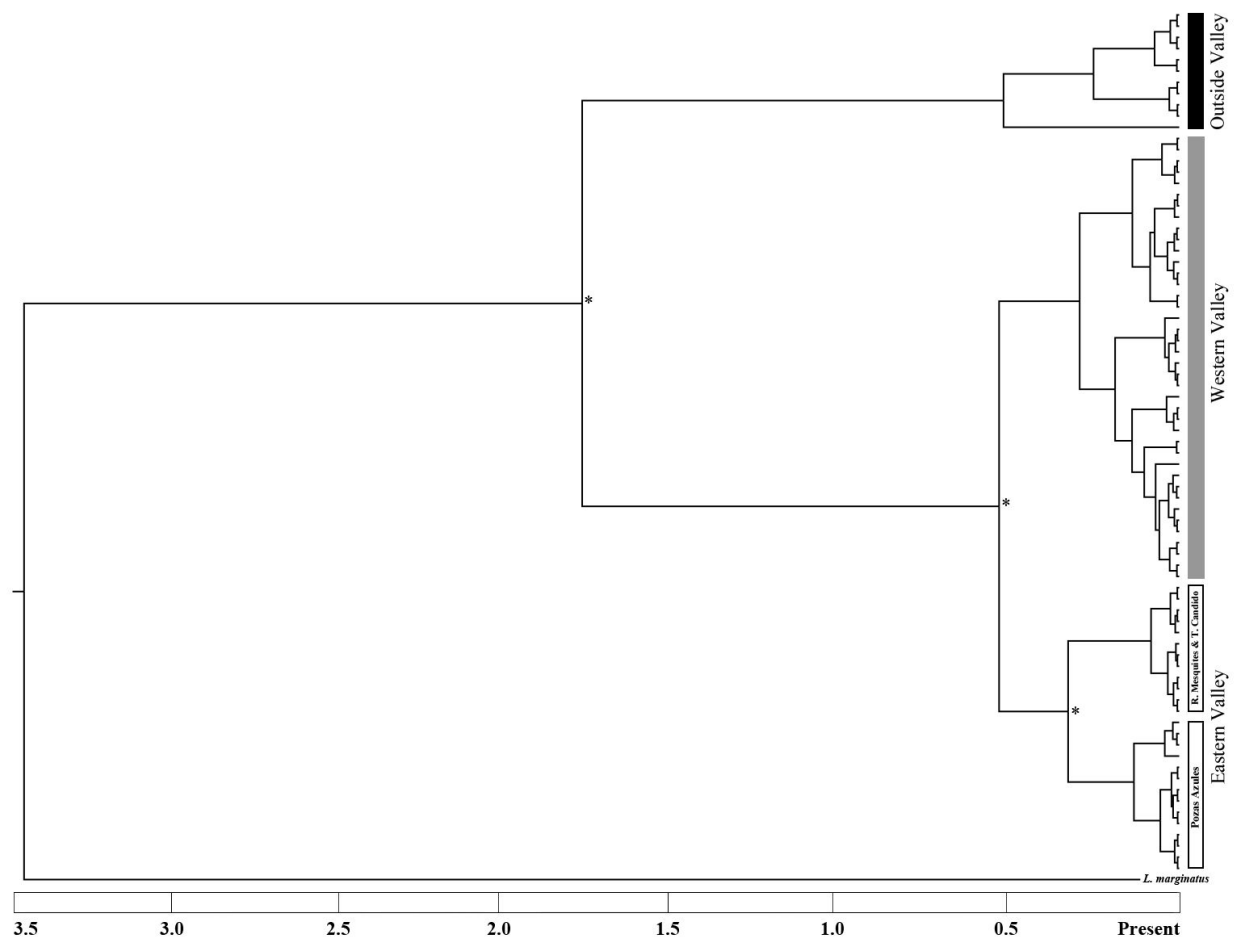
Bayesian gene tree estimated from 77 individuals using 2839bp (ND2: 1047, Cytb: 1140 and COI: 652) of the mitochondrial genome. Geographically isolated regions within the valley are highlighted with shading for emphasis. An * denotes posterior probability support greater than 0.98.

### Population Structure

Following previous studies [6,12,23] regions were initially defined based on geographic boundaries based on the position of the sampling sites relative to the Sierra (Figure 1). Pozas Azules, at the far southeastern edge of the valley, was defined as a unique region based on its geographic isolation. The Rio Mesquites and Tio Candido along the eastern edge of the Sierra were grouped to form the “Eastern” region based on the genetic structure of other organisms (Carson & Dowling, 2006; Chaves-Campos et al., 2010). Pozas Bonita and Juan Santos were the locations sampled along the western side of the Sierra and make up the “Western” region. Because of their isolation from other bodies of water and distance from one another, these sites were initially treated as independent regions. In order to evaluate population structure, we performed an Analysis of Molecular Variance (AMOVA) on these four regions (Pozas Azules, Eastern, Juan Santos and Poza Bonita) using ARLEQUIN 3.5 to examine differences among the sampled regions within the valley [24].

**Figure 4:**
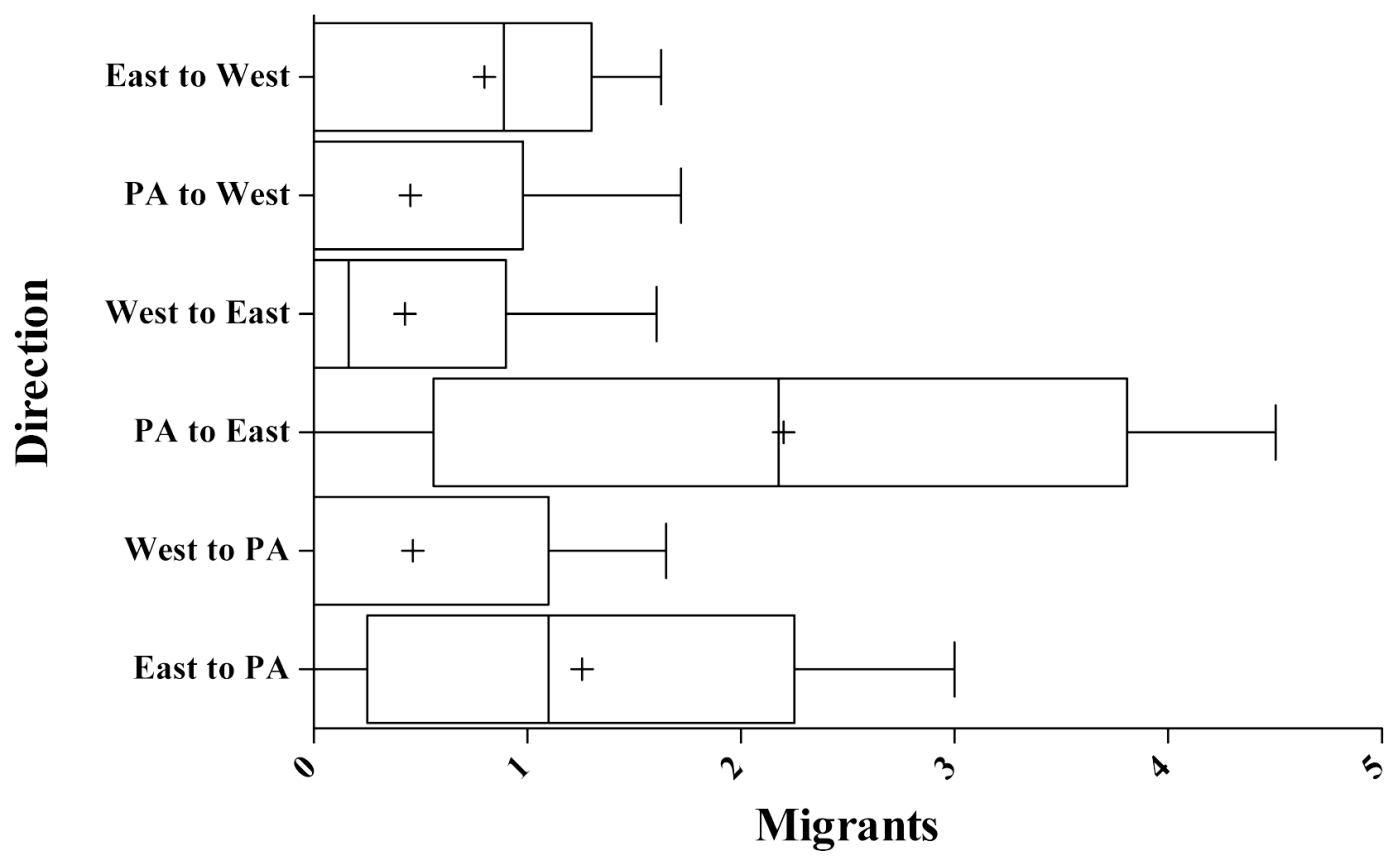
Estimates of gene flow based on Bayesian inferences of migration rates and population sizes. M is the estimated migration rate, scaled for the appropriate mutation rate between the different population clusters. Box plots represent values from the lower (25%) to upper (75%) quartiles with the median value marked as a +. The lines extend from the 2.5% to 97.5% percentiles.

### Phylogeographic Analysis

For the phylogeographic analyses, sequences were aligned with MUSCLE, and a haplotype network was constructed using the median joining method [25] implemented in the program Network version 4.611 [26,27]. Unique haplotypes were coded according to the regions mentioned above, and jMODELTEST 2.0 was used to choose the best fitting, least-parameter rich model of sequence evolution based on Bayesian Information Criterion [28]. The program BEAST v 1.7.4 was then used to simultaneously estimate a gene tree and the divergence of haplotypes among regions [29]. We partitioned and applied the appropriate model of molecular evolution to each gene (HKY+G for COI and ND2, and GTR+G for Cytb). An uncorrelated log normal relaxed clock was used to estimate divergence times based on a fossil-calibrated split between *L. megalotis* and *L. marginatus* of 1.72 ± 0.83 million years [30]. The relaxed clock, uncorrelated lognormal model allows simultaneous estimation of phylogeny and divergence times [31]. Two primary analyses were conducted. The first constrained the individuals from Cuatro Ciénegas to be a monophyletic clade. The second allowed all individuals to be assigned to any particular clade during the analysis. Each analysis was run for 10,000,000 generations starting with a random starting tree, constant size coalescent prior, and a burn-in of at least 1,000,000 [32,33]. The analysis was repeated three times to confirm the robustness of the topology and divergence time estimates [33].

For our two models examining population structure in Cuatro Ciénegas, the BEAST output was inspected and analyses of Bayes Factors were performed using Tracer 1.5. This allowed us to examine the posterior distributions, to check for convergence, and to confirm that the effective sample size for each parameter exceeded 200 [33]. Posterior probabilities and the “maximum clade credibility tree” were calculated using TreeAnnotator 1.5.4 [33]. 

### Gene Flow Analysis

The grouping pattern and splitting order of divergent populations recovered in the BEAST gene tree were used to estimate gene flow under the coalescent in MIGRATE-N 3.5.1 [34-36]. Four migration models were tested: (1) bi-directional gene flow between all 3 well supported clades (Pozas Azules, Eastern and Western) recovered from the phylogeographic analysis, (2) two populations divided strictly by the Sierra de San Marcos (Eastern and Western), (3) a split between Pozas Azules and the remainder of the valley, (4) a panmictic model assuming open gene flow between all populations. Our MIGRATE-N 3.2.6 analyses were implemented with default parameters except for modifications to run-length, heating, and relative mutation rate that were specific to the different migration models. To calculate marginal likelihoods for the model comparisons, we used a heating scheme of 1.00, 1.50, 3.00, and 1,000,000.00. After the runs were completed, results of each model were compared using Bayes Factors calculated from the model probabilities as described in MIGRATE-N [36,37]. 

**Table 2:** Summary of genetic differentiation by region of *Lepomis mega loti*.

	**Pozas Azules**	**Eastern Valley**	**Juan Santos**	**Poza Bonita**
**Pozas Azules**		<0.0001	<0.0001	<0.0001
**Eastern Valley**	0.78		<0.0001	0.04
**Juan Santos**	0.92	0.58		<0.0001
**Poza Bonita**	0.88	0.71	0.41	

Pairwise *F*_*ST*_ values are presented below the diagonal. The corresponding *P*-values of significance from zero are presented above the diagonal.

## Results

### Population Structure

Mitochondrial haplotype diversity among populations of *L. megalotis* was substantial. A total of 26 unique haplotypes were recovered from the 77 individuals sampled across the entire valley (Figure 2). Distances among these unique haplotypes ranged from 0.1% to 7.4%. The AMOVA (Table 2) showed that the haplotypes were not homogeneously distributed in the Cuatro Ciénegas valley: 74% of sequence variation is due to differences among regions (Pozas Azules, Eastern Valley, Juan Santos and Poza Bonita), while the remaining 26% is due to differences found within those regions (*F*_*ST*_ = 0.92, *P* < 0.001). Pairwise *F*_*ST*_ values between all of the regions were high (>0.71), and most were significantly different from zero. The exceptions were comparisons among the Eastern Valley (Río mesquites and Tio Candido), Juan Santos and Poza Bonita, which had lower *F*_*ST*_ values (<0.58). 

The haplotype network analysis recovered several unique haplotype clusters within Cuatro Cienegas that largely fell along sampling localities. These were: 1) Poza Bonita 2) Western Valley (Juan Santos and Poza Bonita). 3) Río Mesquites and Tío Candido (Eastern Valley), as well as 4) Pozas Azules in the southeastern lobe of the valley. 

### Phylogeographic Analysis

The BEAST analyses identified major phylogeographic structure within the valley. There was a clear division between populations within the valley and those found outside the valley with a posterior probability support of 1.0 and an estimated divergence time of 1.75 million years (Figure 3). All of the individuals within the valley share a most recent common ancestor. However, within the Cuatro Ciénegas valley there was support for splitting *L. megalotis* into three distinct phylo-groups. The timing of the oldest split recovered suggests that the Eastern and Western populations (Poza Bonita and Juan Santos) of the valley diverged approximately 0.55 million years ago (posterior probability support of 0.99). Within the Eastern valley clade, the individuals from Tio Candido and Rio Mesquites clustered together. These populations were inferred to have diverged from the Pozas Azules clade approximately 0.40 million years ago (posterior probability support 0.98). The Juan Santos and Poza Bonita groups form a distinct clade on the Western side of the valley that is well supported. There also was a phylogeographic split between Juan Santos and Poza Bonita individuals based on the haplotype network and AMOVA results. However, the posterior probability support for this divergence was low (0.64). While most of the individuals were found in only one geographically defined clade, three individuals from the Eastern Valley did fall out within the Pozas Azules clade. Four individuals from Pozas Azules also fell out in the primarily Eastern clade. There was also a few shared haplotypes between the Poza Bonita clade and Juan Santos clade. Two Juan Santos individuals grouped with Poza Bonita and one individual from Poza Bonita grouped with the primarily Juan Santos haplotypes. However, it is important to note that no haplotypes were shared between the Poza Azules + Eastern clade and the Western Clade. Additionally, Bayes factor analyses supported the monophyly of the Cuatro Ciénegas valley clade with a log_10_ Bayes factor value of 3.2 indicating monophyly is highly (1000) times more likely than non-monophyly.

**Table 3:** Comparison of gene flow models using Bayes Factors.

**Model**	**Structure**	**Bezier lML**	**Harmonic lML**	**Probability**
**A**	3 Pops	-3022	-3241	0.912
**B**	2 Pops (E&W)	-3221	-3065	0.058
**C**	2 Pops (V&PA)	-3111	-2781	0.030
**D**	Panmictic	-3267	-2930	0.000

Between 3 populations, eastern valley, western valley and Pozas Azules. Eastern valley populations (E) and western valley populations (W), between Pozas Azules (PA) and the rest of the valley (V) and a complete panmictic single population. Estimates of model probabilities derived from using summarized log marginal likelihoods and natural log Bayes factors. Model of the highest probability is reported in bold. Harmonic means are reported but were not used in the analysis, as the variance in the harmonic mean is generally too large to recover the best model.

### Gene Flow

The MIGRATE-N 3.5.1 [35] results suggest that levels of gene flow were overall fairly minor across the valley with most populations experience less than 1 migrant per generation (Figure 4). The highest levels of inferred migration were found between Pozas Azules and the Eastern populations. However, the median levels of migration between even these two populations were still quite low with approximately 2 migrants between these populations per generation. Overall, the gene flow analysis supports high levels of genetic structure and low levels of migration. With a probability of 0.912 (Table 3), the Bayes factor analyses suggested that among the models tested, the model defining three distinct populations (Pozas Azules, Eastern Valley and Western Valley) is the best-supported characterization of *L. megalotis* population subdivision within Cuatro Ciénegas. 

## Discussion

The findings presented here suggest that the populations of *L. megalotis* within the valley are highly divergent from *L. megalotis* populations found outside Cuatro Ciénegas. The level of divergence observed between populations found inside the valley and outside the valley leads us to infer that *L. megalotis* did not invade the valley in the past 0.5 million years. Populations of *L. megalotis* also exhibit a substantial amount of genetic differentiation and little gene flow among the various populations examined within the valley. The observed genetic differentiation has a number of conservation implications for this fish and its unique habitat [38,39].

All individuals from Cuatro Ciénegas form a monophyletic clade that likely split from the *L. megalotis* populations outside the valley approximately 1.0 - 2.3 million years ago. This substantial divergence from other *L. megalotis* populations mirrors what has been found in other species present both inside the valley and in the Río Salado, the closest watershed to Cuatro Ciénegas [6]. This result also is consistent with suggestions by Smith (1984) who reported that the deserts of North America experienced a cycle of heavy precipitation between 1.3 and 3.2 Myr ago that might have led to connections between Cuatro Ciénegas and external drainages. Importantly, the timeframe of genetic divergence within *L. megalotis* suggests that the populations of *L. megalotis* in the Cuatro Ciénegas basin are likely native to this region and evolutionarily distinct from populations found outside the valley. More focused conservation efforts for this distinctive Cuatro Cienegas lineage of sunfish should be considered [40].

We also found that populations within the valley show high levels of phylogeographic structure and relatively ancient population divergence. Our molecular clock estimates indicate that the Pozas Azules region likely split from other populations in the Eastern region approximately 400,000 years ago. Additionally, the populations from the Poza Azules + Eastern region of the valley split from the Western region of the valley approximately 550,000 years ago. These results mirror what has been found for a number of other taxa that show very high levels of divergence between the regions of the Cuatro Ciénegas valley found on either side of the Sierra de San Marcos [6,8,12,13]. Additionally, although the eastern lobe of the valley is currently receiving a substantial amount of conservation attention and protection of habitats, the western lobe of the valley is not [9]. If it is a management priority to preserve the unique fauna of Cuatro Ciénegas, the genetically distinct lineages of organisms and the habitats on the western lobe of the valley should receive greater conservation consideration [40].

Despite the genetic isolation between the three major population clusters of the valley, we did infer that there are low levels of gene flow among some locations. These low levels of gene flow could be signatures of more recent aquatic corridors that existed during wet cycles of the Holocene around approximately 11,000 years ago [41]. Another possible explanation are rare flood events, such as hurricanes, which can flood much of the valley floor and could facilitate movement among otherwise disjunct locations [6]. It is also possible that the canal systems built within the last hundred years that connects the pools and streams near the Rio Mesquites and Pozas Azules could be allowing gene flow between long isolated regions [6,9]. This canal-mediated mixing is supported by the fact that a small number of haplotypes from the Eastern valley region were recovered in the Pozas Azules region and vice versa despite otherwise substantial divergence between these two populations (Figure 2). Other studies have recovered similar patterns [6] suggesting that these canal systems could be facilitating genetic mixing of evolutionarily distinct populations of aquatic organisms within the valley [42].

## Conclusion

Most of Cuatro Ciénegas is currently managed as a single conservation unit. Our results, combined with other phylogeographic studies within the valley, indicate that Cuatro Ciénegas is made up of several historically independent regions that are inhabited by distinctive genetic lineages. Management efforts should begin to account for how distinctive the faunas of the different lobes of the valley are. Populations of *L. megalotis* within the valley are also quite genetically distinct from populations found outside the valley, and this should reinforce the general recognition that the valley contains a highly unique vertebrate fauna [14,43-45]. We also found evidence consistent with human-mediated habitat changes in the form of canals putting evolutionarily unique populations of *Lepomis megalotis* at risk [46,47]. The continued increases in water use in and around Cuatro Ciénegas could result in the irrevocable loss of one of North America’s most distinctive faunas whose genetic differentiation we are only now coming to fully appreciate.
